# HOXA13 is a potential GBM diagnostic marker and promotes glioma invasion by activating the Wnt and TGF-β pathways

**DOI:** 10.18632/oncotarget.4813

**Published:** 2015-07-31

**Authors:** Ran Duan, Lei Han, Qixue Wang, Jianwei Wei, Luyue Chen, Jianning Zhang, Chunsheng Kang, Lei Wang

**Affiliations:** ^1^ Department of Neurosurgery, Beijing Tian Tan Hospital, Capital Medical University, Beijing, China; ^2^ Laboratory of Neuro-Oncology, Tianjin Neurological Institute, Tianjin, China; ^3^ Department of Neurosurgery, Tianjin Medical University General Hospital, Tianjin, China; ^4^ Key Laboratory of Neurotrauma, Variation, and Regeneration, Ministry of Education and Tianjin Municipal Government, Tianjin, China; ^5^ Chinese Glioma Cooperative Group (CGCG), Beijing, China; ^6^ China National Clinical Research Center for Neurological Diseases, Beijing, China

**Keywords:** Homeobox (HOX) gene, HOXA13, glioma, SMAD, epithelial-to-mesenchymal transition (EMT)

## Abstract

Homeobox (HOX) genes, including HOXA13, are involved in human cancer. We found that HOXA13 expression was associated with glioma grade and prognosis. Bioinformatics analysis revealed that most of the HOXA13-associated genes were enriched in cancer-related signaling pathways and mainly involved in the regulation of transcription. We transfected four glioma cell lines with Lenti-si HOXA13. HOXA13 increased cell proliferation and invasion and inhibited apoptosis. HOXA13 decreased β-catenin, phospho-SMAD2, and phospho-SMAD3 in the nucleus and increased phospho-β-catenin in the cytoplasm. Furthermore, downregulation of HOXA13 in orthotopic tumors decreased tumor growth. We suggest that HOXA13 promotes glioma progression in part via Wnt- and TGF-β-induced EMT and is a potential diagnostic biomarker for glioblastoma and an independent prognostic factor in high-grade glioma.

## INTRODUCTION

The Homeobox genes (Hox genes), which were firstly discovered in *Drosophila*, are characterized by the existence of a recognizable 183-base pair DNA sequence that encodes a highly conserved 61-amino acid peptide known as the homeodomain [1]. Further studies demonstrated that this gene family orchestrates cell differentiation during embryonic development in many different lineages and developmental pathways, [2, 3]. During development, Hox gene expression controls the identity of body regions according to the rules of spatio-temporal colinearity [4]. In humans, a total of 39 coding HOX genes reside in four separate clusters that are located on four different chromosomes (HOXA at 7p15.3, HOXB at 17p21.3, HOXC at 12q13.3, and HOXD at 2q31) [5]. The HOX genes encode homeoproteins, which are small proteins that act as transcription factors during normal embryonic development and are critical for proper anterior-posterior axis formation in embryos; the expression of HOX genes in a given tissue differs depending on the tissue and time of development [6, 7].

**Figure 1:**
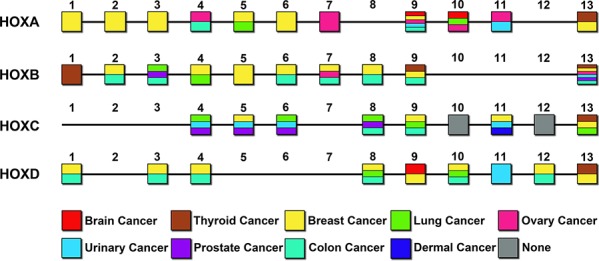
Expression of HOX genes in different solid tumors The abnormal expression of HOX genes (coding genes) in various solid tumors is shown in respective gene sites. The different colors correspond to nine common solid tumors.

Recent studies revealed that the HOX genes are involved in the development of several human cancers (Figure 1). Abnormal expression of HOX genes is associated with disease progression and predicts outcome. Besides, the expression of the newly described lncRNA HOTTIP (HOXA distal transcript antisense RNA, consistent with its genomic location 5′ to HOXA13), lncRNA HIT [HOXA transcript induced by TGF-β, located between HOXA13 and HOXA11-AS (HOXA11 antisense RNA)] is associated with disease progression and predicts outcome in hepatocellular carcinoma patients [8–10]. Several recent reports have also suggested that high expression of HOXA9 [11] and HOXA10 [12] is an indicator of poor prognosis in GBM patients [13]. In addition, our previous research demonstrated that HOTAIR (HOX transcript antisense RNA), a noncoding RNA at the distal tip of the HOXC cluster, is a negative prognostic factor for glioma patients and that its expression could promote cell cycle progression in gliomas as a result of the binding of its 5′ domain to the PRC2 complex [14, 15]. Taken together, a substantial body of scientific evidence indicates that the expression of HOX genes that are critical for normal embryonic development is aberrant in and contributes to carcinogenesis.

Gliomas are a heterogeneous group of neoplasias that account for the majority of primary tumors of the central nervous system; glioblastoma multiforme (GBM) is by far the most common and malignant subtype of glioma and carries a grim prognosis. Despite advances in combination treatments consisting of radiation and chemotherapy following surgical resection, the prognosis of patients afflicted with this disease remains dismal, with a median survival after diagnosis of approximately 15 months [16, 17]. Therefore, it is necessary to identify novel biomarkers that are specific to the stages of glioma or to the susceptibility of gliomas to anti-cancer agents; these tools would help to improve disease prediction in these patients.

HOXA13 is situated at the 5′end of the HOXA locus between HOXA11-AS (HOXA11 antisense RNA lncRNA) and HOTTIP (Two lncRNA of HOXA locus). Recent studies demonstrated that HOXA13 was involved in carcinogenesis and the promotion of tumor growth. Luca Quagliata [8] and Ting-Ting Pan [18] reported that abnormal HOXA13 expression was correlated with hepatocellular carcinoma (HCC) and that HOXA13 overexpression played an important role in HCC progression, metastasis, and poor prognosis. Another study suggested that HOXA13 was involved in carcinogenesis and the promotion of prostate cancer. Although HOXA13 is altered in a wide variety of human tumors, its role in glioma remains unclear.

Here we detected the mRNA and protein expression of the 5′ HOXA genes and found the correlation between HOXA13 with tumor grade, poor prognosis in glioma. We also elucidated the involvement of the HOXA13 in glioma progression and uncovered the oncogene role of HOXA13; HOXA13 promotes GBM development by participating in the regulation of EMT due to the activation of a Wnt/beta-catenin and TGF-β signaling pathway. In addition, HOXA13 also inhibited glioma cell growth in glioma cell lines and a xenograft model. Based on our research, HOXA13 may be a novel diagnostic and prognostic marker for HGG patients.

## RESULTS

### Protein and mRNA expression of 5′ HOXA genes are associated with tumor grade in human glioma specimens

**Figure 2:**
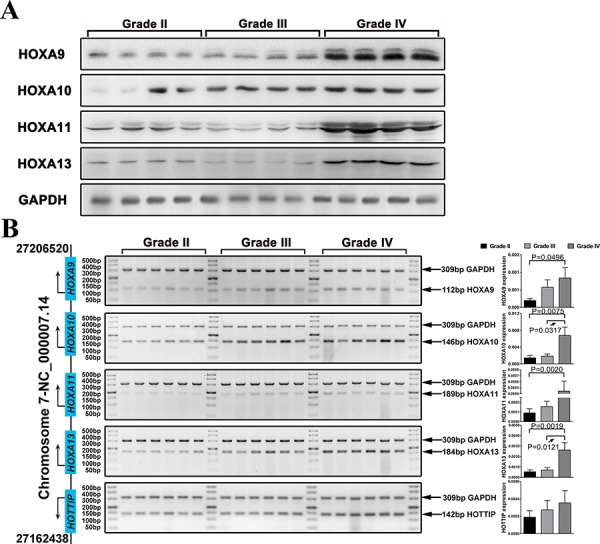
The expression of 5′ HOXA genes is increased in high grade glioma **A.** Western blot analysis was used to examine the expression of HOXA9, HOXA10, HOXA11, and HOXA13 in glioma samples. GAPDH was used as a loading control. **B.** HOXA9, HOXA10, HOXA11, and HOXA13 mRNA expression levels in 64 glioma cases were analyzed by qRT-PCR. The reaction products were electrophoresed through a 3.0% agarose gel that was stained with ethidium bromide and photographed.

In order to explore the role of the 5′ HOXA genes in gliomas, we first measured the expression of HOXA9, HOXA10, HOXA11, and HOXA13 protein in 66 gliomas specimens of different grades. By western-blot analysis, HOXA9, HOXA11, and HOXA13 were dramatically up-regulated in GBM, and HOXA10 merely showed a slight increase in high-grade glioma (Figure 2A). Furthermore, the mRNA expression of HOXA9, HOXA10, HOXA11, HOXA13, and HOTTIP was assayed by qPCR. The expression of HOX10 and HOXA13 mRNA was increased in GBM specimens compared to lower-grade glioma. Accordingly, HOXA9 and HOXA11 expression also increased from lower-grade glioma to high-grade glioma. In addition, high-grade gliomas had high transcriptional levels of HOTTIP, which is a lncRNA that is situated at the 5′ end of the HOXA locus, but this difference was not statistically significant (Figure 2B).

### HOXA13 is up-regulated in high-grade gliomas

**Figure 3:**
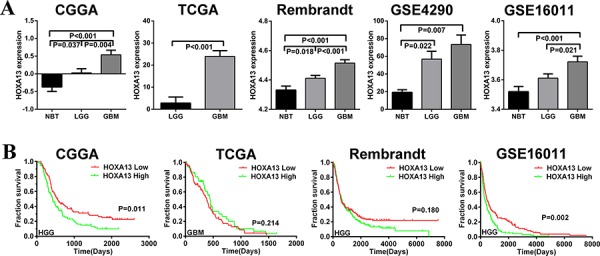
HOXA13 mRNA expression is increased in GBM, and high expression of HOXA13 is associated with poor prognoses in some glioma cases **A.** The expression levels of HOXA13 were analyzed in glioma tissues of the CGGA, TCGA, Rembrandt, GSE4290 and GSE16011 glioma datasets. **B.** Kaplan-Meier survival curve analysis of the CGGA, TCGA, Rembrandt and GSE16011 glioma datasets indicated that some HGG patients with lower HOXA13 expression showed prolonged survival compared to patients with high levels of HOXA13.

Previous studies have shown that the expression level of HOXA13 differs in several human solid tumors relative to normal tissues. We analyzed glioma samples from patients with glioma in the CGGA cohort. Among the 306 samples, 122 were diagnosed as WHO grade II gliomas, 51 as grade III, 128 as grade IV gliomas, and 5 as normal brain tumor (NBT). The expression of HOXA13 was increased in high-grade gliomas compared to low-grade gliomas and NBT (*P* < 0.001). The expression of HOXA13 was also significantly up-regulated in lower-grade gliomas compared to NBT (*p* = 0.037). In order to avoid deviation resulting from mono-centric data, we also analyzed samples from TCGA, REMBRANDT, GSE16011, and GSE4290. We confirmed that HOXA13 is significantly overexpressed in HGG samples compared to LGG and/or NBT samples (Figure 3A). To determine whether HOXA13 expression levels determine the clinical progression or outcome of glioma patients, we examined the overall survival rates of the high-grade glioma patients (samples from CGGA, REMBRANDT and GSE16011) and GBM patients (samples from TCGA) using Kaplan-Maier analysis and log-rank comparison. High HOXA13 expression was associated with a decreased survival period, but this difference was not statistically significant in some samples (Figure 3B).

### HOXA13 is an independent prognostic factor in HGG patients

High expression of HOXA13 was associated with overall survival in the CGGA HGG samples. We then conducted univariate Cox regression analysis using clinical and genetic variables for 179 patients from the CGGA cohort and found that high expression of HOXA13, age older than the median (median = 42 y), and IDH1 mutation were statistically associated with overall survival (Table 1 & Table 2). We then evaluated the factors that contributed to overall survival using a multivariate Cox proportional hazards model. Both high HOXA13 expression and IDH1 mutations correlated independently with overall survival (HR = 1.485, *P* = 0.025; HR = 0.523, *P* = 0.002, respectively) when considering age at diagnosis and Ki-67 (*P* < 0.05, univariate Cox regression analysis).

**Table 1:** Clinical and molecular pathology features of the high-grade gliomas samples with differential HOXA13 expression

Variable	Low HOXA13	High HOXA13	*P* value
Gender (F/M)	38/51	32/58	0.328
Age at diagnosis (years)	44.39 ± 12.51	47.03 ± 12.22	0.155
Overall survival (days)	808.28 ± 673.51	600.13 ± 521.29	0.023
IDH1 mutation(no mutation/mutation)	65/24	64/26	0.774
MGMT (LOW/HIGH/ND)	33/49/7	29/50/11	0.645
Ki67 (LOW/HIGH/ND)	37/45/7	30/49/11	0.358
MMP9 (LOW/HIGH/ND)	17/64/8	12/63/15	0.424
PTEN (LOW/HIGH/ND)	55/76/8	7/68/15	0.459
EGFR (LOW/HIGH/ND)	37/45/7	29/50/11	0.278
P53 (LOW/HIGH/ND)	28/54/7	26/53/11	0.868

**Table 2:** Cox hazards regression analyses of clinicopathologic factors and HOXA13 expression in high grade samples

Variable	Univariable Regression		Multivariable Regression	
	HR (95% CI)	*P* value	HR (95% CI)	*P* value
Gender (female vs. male)	0.979 (0.700–1.369)	0.902		
AGE (<42 y vs. ≥ 42 y)	0.652 (0.463–0.917)	0.014	0.799 (0.531–1.142)	0.201
HOXA13 (High vs. Low)	1.515 (1.089–2.107)	0.014	1.485 (1.052–2.098)	0.025
IDH1 (High vs. Low)	0.509 (0.345–0.752)	0.001	0.523 (0.346–0.791)	0.002
MGMT (High vs. Low)	0.944 (0.666–1.338)	0.746		
MMP9 (High vs. Low)	0.780 (0.506–1.201)	0.259		
PTEN (High vs. Low)	0.602 (0.315–1.154)	0.126		
EGFR (High vs. Low)	1.165 (0.820–1.657)	0.394		
P53 (High vs. Low)	0.882 (0.613–1.269)	0.498		
Ki67 (High vs. Low)	1.511 (1.061–2.153)	0.022	1.492 (1.045–2.131)	0.028

### HOXA13-associated genes are mainly enriched in cancer pathways

**Figure 4:**
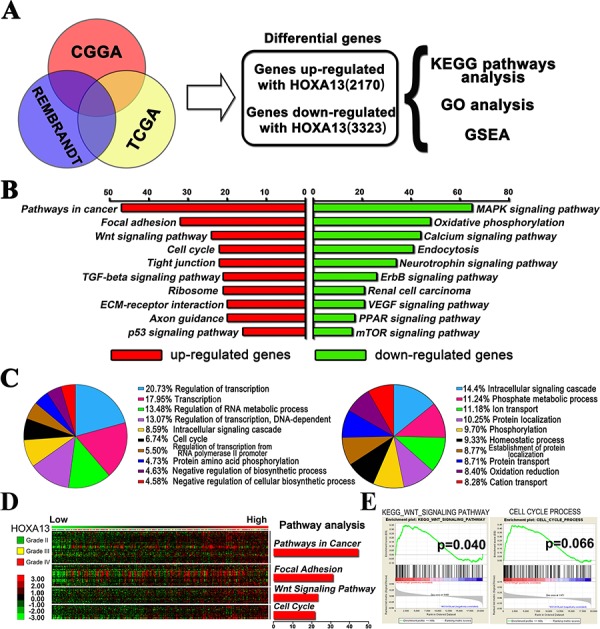
The HOXA13-associated genes were chiefly enriched in cancer related pathway **A.** Correlation analysis performed in the CGGA, TCGA, and Rembrandt glioma samples. HOXA13 associated genes from overlapping CGGA, TCGA and Rembrandt databases were analyzed with KEEG pathway analysis, gene ontology analysis and gene set enrichment analysis (GSEA). **B.** Enrichment analysis results for pathways analysis. These data were obtained from the KEGG database; the red color corresponds to the up-regulated genes, and the green color corresponds to the down-regulated genes. **C.** Biological processes enrichment results of two sets of differential genes. This information was retrieved from the GO database. The orders of biological processes listed in the circle are based on their enriched number. **D.** Correlation analysis and Pathway analysis performed in 301 glioma samples with mRNA expression. A heat map of relative expression of HOXA13-associated genes in glioma tissues sorted by level of HOXA13 expression **E.** GSEA analysis of gene ontology terms showed that there was enriched expression of gene sets involved in Wnt signaling pathway and cell cycle progression in glioma patients.

A Pearson Correlation analysis was carried out using Matlab software and to identify target genes that were associated with HOXA13 in the CGGA, TCGA, and Rembrandt glioma samples. These genes were overlapped and processed using the DAVID Web tool (http://david.abcc.ncifcrf.gov/home.jsp) to identify associations between these genes and specific GO functional categories via KEGG pathway analysis and gene set enrichment analysis (GSEA). A total of 2, 170 up-regulated genes and 3, 323 down-regulated genes were identified (Figure 4A). As illustrated in Figure 4B & 4D, the up-regulated gene expression profiles were more strongly enriched in pathways related to cancer, focal adhesion, Wnt signaling pathways, and the cell cycle. The up-regulated pathways (focal adhesion, Wnt signaling, cell cycle, and TGF-β) suggested that high HOXA13 expression may contribute to the progression of glioma due to cell invasion, adhesion, and migration. Furthermore, GSEA was also used to evaluate the pathways that were differentially expressed in patients with high levels of HOXA13 expression and those with low levels of HOXA13 expression. GSEA analysis revealed that HOXA13 activates genes that are primarily associated with the Wnt signaling pathway and cell cycle progression (Figure 4E). In addition, the resulting GO terms were ranked from the largest to smallest number of genes, and the top ten terms are shown in the pie chart. According to the chart, the HOXA13-associated genes were mainly involved in regulation of transcription (Figure 4C). Overall, these differential pathways and functional analysis implied that HOXA13 may enhance glioma invasion and migration, and this process is carried out through several important cancer-related pathways.

### Lenti-si HOXA13 suppresses the expression of HOXA13 in the nucleus and affects glioma cells *in vitro*

**Figure 5:**
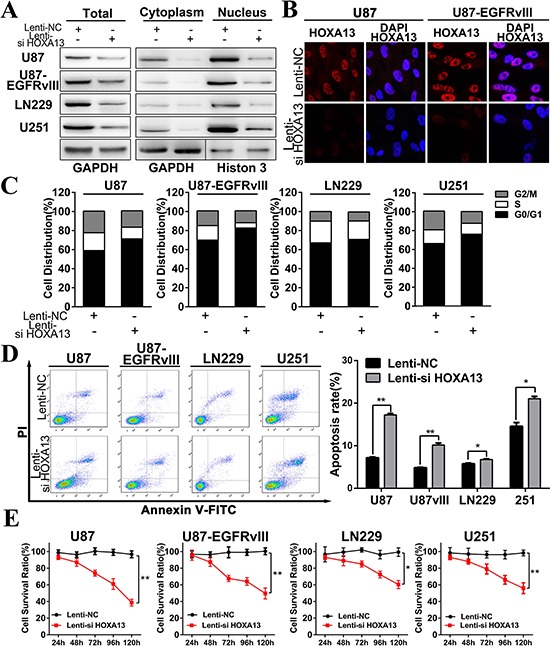
HOXA13 siRNA inhibits proliferation, regulates cell cycle progression, and induces apoptosis in GBM cells *in vitro* **A.** Western blot assays of HOXA13 protein expression level of U87 and U87 EGFRvIII in both the nucleus and cytosol after transfection with a Lenti-si HOXA13 construct. GAPDH was used as a loading control. **B.** HOXA13 expression levels and subcellular location were confirmed using a confocal microscope (1,000×). **C.** Flow cytometry was performed to examine the cell-cycle in U87, U87 EGFRvIII, LN229, and U251 cells after treatment with Lenti-si HOXA13. The percent of cells in the G0/G1, S, and G2/M phases were measured in four cell lines. **D.** Annexin V-PI assays indicated greater levels of apoptosis in the Lenti-si HOXA13-treated group compared to the Lenti-NC-treated group. **E.** Proliferation rates of glioma cells infected with a Lenti-si HOXA13 were measured by MTT assays. All data are represented as the mean +/− SEM; *p < 0.05, **p < 0.01.

Our evidence suggested that HOXA13 was more than a simple transcription factor and implicated HOXA13 in glioma development and progression. We therefore constructed a lentivirus containing a siRNA sequence targeting HOXA13 to verify whether HOXA13 plays a potentially functional role in glioma. After 48 h of infection, HOXA13 protein expression levels in U87, U87-EGFRvIII, LN229, and U251 were decreased, and HOXA13 expression was mainly restricted to the nuclear protein fraction (Figure 5A). To further confirm the subcellular localization of HOXA13 in gliomas, confocal microscopy was used to observe glioma cells. As illustrated in (Figure 5B), confocal microscopy showed HOXA13 staining to be attenuated in the nuclei of Lenti-si HOXA13 transfected glioma cells.

**Figure 6:**
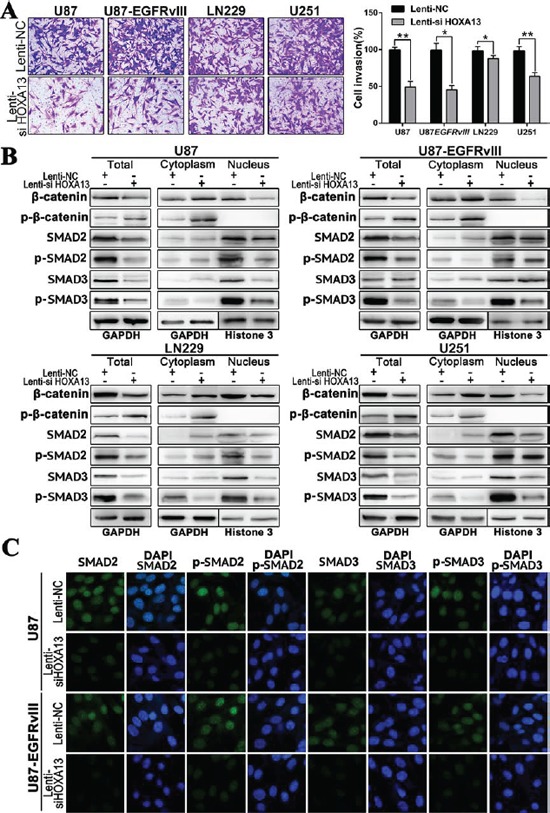
The suppression of HOXA13 inhibits invasion via the Wnt and TFG-β pathways in GBM cells **A.** Transwell assay was used to examine the invasive ability of U87, U87EGFRvIII, LN229 and U251 cell lines after transfection with Lenti-si HOXA13 and Lenti-NC; **B.** The expression of Wnt- and TGF-β pathway-associated markers and their distribution in cytoplasm and nucleus were tested after suppressing HOXA13 in four glioma cell lines. **C.** SMAD2, p-SMAD2, SMAD3 and p-SMAD3 expression and subcellular location were confirmed by confocal microscopy.

To study the biological implication of HOXA13 in glioma, we performed functional assays to determine HOXA13′s influence on glioma cell proliferation and invasion (Figure 5E & 6A). Decreased HOXA13 expression in U87, U87-EGFRvIII, LN229, and U251 inhibited the proliferation of these four cell lines. Furthermore, Annexin-V/PI staining was conducted to quantify the apoptosis induced by the Lenti-si HOXA13 construct. Compared to the Lenti-NC group, the Lenti-si HOXA13 treatment caused more apoptosis and increased the apoptotic rates in the four glioma cell lines (Figure 5D). Moreover, flow cytometry analysis showed that the cell cycle was blocked in the G0-G1 phase as a result of decreased HOXA13 (Figure 5C). On the basis of the above findings, we conclude that HOXA13 is a potent enhancer of tumor cell growth and duplication *in vitro*. The above observations from the functional experiments were previously unknown, implying that the underlying mechanism need to be explored further.

### The down-regulation of HOXA13 expression inhibits glioma cell invasion by regulating the TGF-β signaling pathway *in vitro*

The down-regulation of HOXA13 in glioma cells resulted in a marked decrease in cell invasion in Transwell assays compared to the control cell type, and this effect was more significant in the U87 and U87-EGFRvIII cell lines (Figure 6A). The above results demonstrated that decreased HOXA13 expression inhibited the growth and proliferation of glioma cells. However, the underlying mechanism is still unknown. Beta-catenin is a critical factor in the Wnt/beta-catenin signaling pathway. Our bioinformatics analysis implied that the genes that were positively correlated with HOXA13 were enriched for Wnt pathway factors, so we measured β-catenin and phospho-β-catenin (p-β-catenin) levels by western blotting. The down-regulation of HOXA13 triggered a reduction of nuclear β-catenin in four glioma cell lines, and p-β-catenin was induced by Lenti-HOXA13 in the cytoplasm. However, the cytoplasmic expression of β-catenin was slightly increased. Additionally, the p-β-catenin expression was elevated in the HOXA13 down-regulated cells. These results confirmed that Lenti-HOXA13 suppressed the transcription and translational expression of β-catenin (Figure 6B).

By KEGG analysis, the HOXA13-associated genes were involved in the TGF-β pathway, which is correlated to the EMT process; SMAD2 and SMAD3 were involved in a “Cancer Pathway.” Therefore, we further assayed SMAD2, SMAD3, and their respective phosphorylated forms by western blotting. As shown in Figure 6B, although the levels of SMAD2 and SMAD3 decreased slightly, the levels of hyperphosphorylated SMAD2 and SMAD3 (p-SMAD2 and p-SMAD3) were markedly inhibited in the nucleus. We next performed immunofluorescence staining to evaluate changes in SMAD2, SMAD3, p-SMAD2, and p-SMAD3 expression as a result of HOXA13 depletion.

Confocal microscopy analysis suggested that Lenti-si HOXA13 treatment of the U87 and U87-EGFRvIII cell lines resulted in decreased nuclear expression of p-SMAD2 and p-SMAD3, which were mainly located in the nucleus (Figure 6C). This was observed as significantly less fluorescence signal intensity in the nucleus compared to the Lenti-NC groups, suggesting that HOXA13 may contribute to an enhanced invasion ability of glioma cells dependent on TGF-β-induced EMT.

### HOXA13 knockdown in U87-EGFRvIII cells impedes orthotopic tumor growth *in vivo*

**Figure 7:**
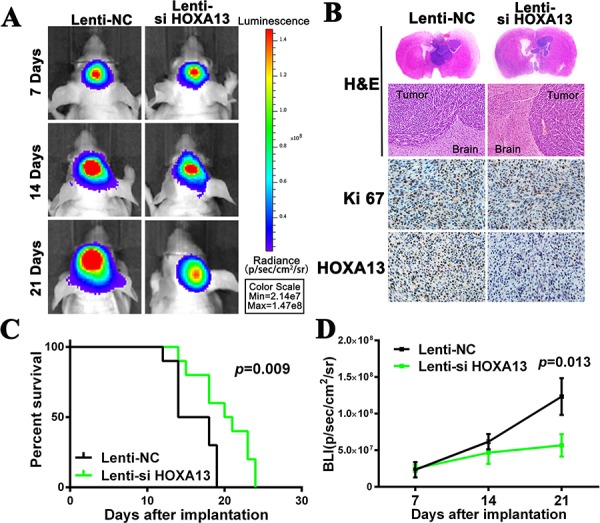
The suppression of HOXA13 inhibits tumor growth and is associated with good prognosis in an intracranial glioma murine xenograft model **A.** U87EGFRvIII cells pretreated with a lentivirus with HOXA13 siRNA or NC siRNA and a lentivirus containing luciferase were implanted in the brains of nude mice, and tumor formation was assessed by bioluminescence imaging. Changes in the bioluminescent signal were measured at days 7, 14 and 21 after implantation. **B.** Tissue slices from representative tumors from the two groups were stained with Hematoxylin-eosin-saffron. The images show representative immunohistochemical staining for Ki67 and HOXA13. **C.** Overall survival was determined by Kaplan-Meier survival curves, and a log-rank test was used to assess the statistical significance of the differences. **D.** Bioluminescence (BLI) was monitored to assess the tumor growth at days 7, 14 and 21. *P < 0.05.

To further verify the role of HOXA13 and to determine the therapeutic potential of depleting HOXA13, we constructed orthotopic mouse models using U87-EGFRvIII cell lines. Prior to implantation, we co-infected U87-EGFRvIII glioma cells with lentiviruses expressing luciferase and a Lenti-NC or Lenti-si HOXA13 for 48 h. In these models, Lenti-si HOXA13 resulted in a significant reduction of the intracranial tumor volume compared to the Lenti-NC groups. *In vivo* imaging analysis of the mice at 7, 14 and 21 days after implantation revealed that the growth of orthotopic tumors was significantly inhibited by the decreased expression of HOXA13 (Figure 7A & 7D). In addition, treating with Lenti-si HOXA13 was associated with significantly longer survival times in the mice (*p* = 0.009) (Figure 7C). Similarly, hematoxylin and eosin staining of the brain tissues resected from the orthotopic mice showed that the Lenti-si HOXA13-treated tumor volume was reduced when compared to the Lenti-NC-treated tumors. Furthermore, IHC showed the decreased expression of Ki-67 and HOXA13, which is consistent with the *in vitro* results (Figure 7B). Altogether, these *in vivo* findings demonstrate that HOXA13 inhibits the proliferation of glioma cells *in vivo*.

## DISCUSSION

Tumorigenesis and embryogenesis share same events such as growth, differentiation and organization of cell populations. Expression of many genes that are critical for normal embryonic development is aberrant in and contributes to carcinogenesis. As a class of remarkable evolutionarily conserved and development-associated genes, HOX genes have been the topic of various cancer-related studies due to their aberrant expression in many solid tumors [9, 12, 13].

Many reports have identified HOXA9 and HOXA10 signatures in GBM, and high expression levels of HOXA9 and HOXA10 were predictive of shorter survival in some high-grade glioma patients [11]. In the present study, we first analyzed the expression of HOXA9, HOXA10, HOXA11, and HOXA13 protein in glioma tissues; we then further analyzed the expression of HOXA9, HOXA10, HOXA11, HOXA13, and HOTTIP mRNA in the same samples. Our results demonstrated a significant increase in the expression of HOXA9-A13 in high-grade gliomas (WHO III and WHO IV). In addition, the expression of HOXA10 and HOXA13 increased in the WHO IV gliomas compared to the WHO II & III gliomas, and the mRNA expression of HOTTIP increased from WHO II gliomas to WHO IV gliomas. Interestingly, the expression of the 5′ HOXA mRNAs was on a similar upward trend in these samples of different grades. These findings imply that aberrant high expression of the HOXA9-HOTTIP genes expression most likely contributes to malignant progression in gliomas and suggests that HOTTIP (as the 5′ HOXA lncRNA) may directly or indirectly control the activation of the four 5′ HOXA-encoding genes in gliomas [8].

HOXA13 plays a crucial role in carcinogenesis [18, 19]. We hypothesized that HOXA13 may be associated with glioma. To test this hypothesis, we analyzed the expression of HOXA13 in several databases. We found that HOXA13 levels were significantly higher in HGG compared with NBT or LGG in all cohorts. In survival analyses, cross-validation from the CGGA and GSE16011 cohorts identified HOXA13 as a significant biomarker in HGG outcome. The up-regulated expression of HOXA13 correlated with poor outcome in HGG patients. Our data indicated that HOXA13 can be used as a potential diagnostic marker and also as a prognostic marker for some HGGs.

To further understand biological significance of HOXA13 in glioma progression, we overlapped the HOXA13-associated genes from three glioma gene expression datasets and conducted bioinformatics analysis. Pathway analysis revealed that a high proportion of the genes that were positively correlated to HOXA13 were enriched in cancer-related pathways, focal adhesion, the Wnt signaling pathway, and the cell cycle. A heat map showed that the candidate genes involved in these four pathways were most significantly altered along increasing tumor grades with the up-regulation of HOXA13. GSEA demonstrated gene set differences between patients with high and low HOXA13 expression; these differences indicated that HOXA13 mainly stimulates genes that are involved in the Wnt signaling pathway and in cell cycle progression in GBM. Furthermore, GO analysis reviewed that HOXA13 associated genes largely regulate transcription in the biological process of glioma progression. Our results demonstrated that HOXA13 induced glioma cell proliferation, cell cycle distribution, and cell invasion and inhibited apoptosis. The same results were observed in a glioma mouse model. However, our results shown that knockdown HOXA13 decreased cell proliferation and caused apoptosis 48 h after transfection. The tumors shown in BLI image were comparable shown at day 7. There are several reasons for this phenomenon. Although proliferation ability of the cells used for transplantation 48 h after transfection may differ according to our cell biological functions assays, the same number of cells (with the same bioluminescence) was injected in nude mice brain. Besides, both groups' cells underwent centrifugation, resuspend, PBS washing, and injection processes in cell preparation; and the cell microenvironment was changed largely after been transplanted in mice brain. Therefore, the cells transplanted in mice brain needed a period of time to restore proliferation ability resulting in a minor changes in cell number at day 7.

Though data analysis generally showed a dysregulation of HOXA13 in different grade gliomas and a potential biological function of HOXA13 in glioma progression, the function of HOXA13 in GBM remained unclear. To further verify the biological behavior of HOXA13 in gliomas, we constructed lentiviral vectors expressing nonsense control or HOXA13 siRNA, and subsequently infected the U87, U87-EGFRvIII (relatively high HOXA13 expression), LN229, and U251 (relatively low HOXA13 expression) cell lines with these lentiviruses. Firstly, we surprisingly observed that HOXA13 protein was located in the nucleus in these four GBM cell lines. This phenomenon was seemingly inconsistent with the results of other studies. Works from Yang Han [19] et al. showed that HOXA13 staining in immunohistochemistry was observed mainly in the nucleus of cancer cells and in the cytoplasm as well in gastric cancer, but our confocal analysis detected virtually all HOXA13 protein in the nuclei of GBM cells. This difference implies that HOXA13 regulation of cancer cell function may be dependent on the location of HOXA13 protein and that HOXA13 in different subcellular localizations seems to have different functions. Our study also suggests that HOXA13 may promote cell proliferation, invasion, and suppress cell apoptosis. Moreover, we further analyzed the cell cycle distribution of the infected cells by PI staining with flow cytometry and found that a reduction in HOXA13 expression induced a G0 /G1 cell cycle arrest. However, there is not any previous study about the mechanisms of HOXA13 in cell cycle regulation in glioma cells. Resent report indicated that downregulated SMAD2 expression could suppress the G1/G0 arrest in Cervical Cancer Cells [20]. Another report reviewed that cell cycle progression was regulated by the SMAD2/SMAD3 pathway, which is consistent with the study that SMAD2 overexpression could promote cervical cancer cell growth by facilitating the G1/S phase transition [21, 22]. In addition, a study demonstrated that HOTTIP knockdown caused G1/G0 arrest by interaction with HOXA13 [23]. Thus, this evidences suggests that HOXA13 knockdown may cause G0/G1 arrest by inhibiting SMAD2/SMAD3 pathway and this process could be regulated by HOTTIP in glioma.

It is well known that the process of epithelial-to-mesenchymal transition (EMT) is associated with tumor progression and metastasis [24]. The SMAD2 and SMAD3 transcription factors lie at the core of the transforming growth factor (TGF) pathway, which promotes tumor invasion and Epithelial-Mesenchymal Transition (EMT) [25, 26]. SMAD proteins are classified into three classes based on their functions: the receptor-regulated SMAD (R-SMAD), the common-mediator SMAD (Co-SMAD), and the inhibitory SMAD (I-SMAD). The phosphorylation of the conserved C-terminal serine residues in the SXS motif of the MH2 domain of the R-SMAD SMAD2/3 drives the activation of the SMAD proteins; the activated R-SMAD proteins then translocate to the nucleus, oligomerize with SMAD4 (Co-SMAD), and regulate the transcription of TGF-β target genes. The SMAD MH2 domain is highly conserved and represents one of the most versatile protein-interacting modules in signal transduction. SMAD MH2 domains interact with several proteins in the nucleus, effecting transcription [27, 28]. Recently, Two studies demonstrated that both lncRNA HIT and coding-RNA HOXB7 play an important role in TGF-β-induced EMT, cell migration, and invasion [10, 29]. Williams confirmed that HOXA13 was able to interact with the MH2 domain of R-SMAD, without SMAD4 [30]. Therefore, HOXA13 may activate TGF-β signaling by interacting with the MH2 domain of SMAD2/3 to regulate the nucleus-cytoplasm distribution of phosphorylated-SMAD2/3 and promote tumor progression. The latest study confirms that HOTTIP/HOXA13 axis promotes pancreatic ductal adenocarcinoma (PDAC) tumorigenesis. In addition, some researcher supposed that HOXA13 was probable to regulate TGF-β signaling by interacting with RUNX3 (runt-related transcription factor 3) mediated by SMADs to promote tumor progression in gastric cancer [19]. In this report, we provided evidence for this hypothesis by demonstrating that GBM cells treated with a HOXA13 siRNA showed decreased expression of p-SMAD2 and p-SMAD3 in nucleus with no significant change in the total SMAD2 and SMAD3 expression levels. This result verifies that HOXA13, SMAD2/p-SMAD2 and SMAD3/p-SMAD3 primarily co-localize in the nucleus in GBM cells and that HOXA13 may stabilize phosphorylated R-SMAD by interacting with the MH2 domain in order to activate the TGF-β signaling pathway. In the basal state, R-SMADs are predominantly localized to the cytoplasm [31]. However, our results revealed that SMAD2/p-SMAD2 and SMAD3/p-SMAD3 are localized to the nucleus in GBM. This phenomenon suggests that the persistent activation of the TGF-β signaling pathway was due to a dysregulation of R-SMAD nucleocytoplasmic shuttling in gliomas and that HOXA13 may be involved in this process, especially in GBM.

Beta-catenin, the core factor of the Wnt/beta-catenin pathway, moves from the cytoplasm into the nucleus, where it forms stabilized complexes with TCF4/LEF to promote Wnt target gene signaling. In the cytoplasm, phosphorylated β-catenin is recognized by the E3 ubiquitin ligase receptor β-TrCP. After ubiquitination, β-catenin is targeted for rapid destruction by the proteasome [32]. Previous studies have demonstrated an overactivation of the Wnt/β-catenin pathway resulting from multiple oncogenes, maintaining the aggressive malignant phenotype of gliomas [33–35]. KEGG analysis and GSEA revealed that HOXA13-associated genes were involved in the regulation of the Wnt signaling pathway, which is in turn associated with the EMT process. These results suggest that HOXA13 can promote migration and invasion via Wnt-induced EMT. In four glioma cell lines, migration and invasion was inhibited by the introduction of a specific lentivirus containing a HOXA13 siRNA, and the β-catenin proteins levels in the nucleus were decreased after treatment with Lenti-si HOXA13. Notably, the expression of β-catenin and its transcriptional activity were dramatically inhibited by downregulation of HOXA13 in GBM cells. Additionally, it is conceivable that a decrease in proliferation and the attenuation of invasion in GBM cells may be explained by HOXA13 depletion and the subsequent inhibition of Wnt/β-catenin- and TGF-β-induced EMT. In summary, high levels of HOXA13 is associated with GBM and poor prognosis. The down-regulation of HOXA13 decreased invasion and metastasis by inhibiting Wnt/β-catenin and TGF-β induced EMT. *In vivo*, inhibition of HOXA13 inhibited tumor growth and prolonged animal survival.

## MATERIALS AND METHODS

### Clinical specimens and bioinformatics

Our clinical data information was obtained from five large gene expression-profiling glioma cohorts. Clinical information from 301 glioma samples and 5 non-brain tumor (NBTs) specimens from the Chinese Glioma Genome Atlas (CGGA, http://www.cgcg.org.cn/) were included in this study. In addition, 66 frozen glioma tumor samples were obtained from the Glioma Center of Beijing Tiantan Hospital (Table 3). This study was approved by the Research Ethics Committee of Tiantan Hospital. Four external independent glioma databases (TCGA, REMBRANDT, GSE16011, and GSE4290) were included. This study was approved by the institutional review boards of all hospitals involved in the study, and written informed consent was obtained from all patients.

**Table 3:** Clinical information of 66 gliomas specimens

Variable	WHO II	WHO III	WHO IV	Total
No.	18	28	20	66
Mean age (range)	43.8 (27–61)	37.5 (23–54)	49.2 (30–71)	42.9 (23–71)
Gender (%)				
male	11 (61.1%)	15 (53.6%)	10 (50.0%)	36 (54.5%)
female	7 (38.9%)	13 (46.4%)	10 (50.0%)	30 (45.5%)

### Cell culture and lentiviral infection

Human GBM cell lines U87, LN229 and U251 were obtained from ATCC (the American Type Culture Collection, Manassas, VA, USA). U87-EGFRvIII GBM cell line, which carries a mutant EGFR, was kindly provided by Prof. Xia Li of the College of Bioinformatics Science and Technology, Harbin Medical University (Harbin, China). All GBM cell lines were routinely cultured at 37°C in a 10% CO2 humidified atmosphere in Dulbecco's modified Eagle medium (DMEM) with 10% fetal bovine serum (FBS, Hyclone). Lentivirus containing HOXA13 siRNA segments (Lenti-si HOXA13, siRNA sequence is 5′-GCGGACAAGUACAUGGAUATT-3′) or negative control sequence (Lenti-NC, negative control sequence is 5′-TTCTCCGAACGTGTCACGT-3′) was obtained from Genepharma (Shanghai, China). U87, U87-EGFRvIII, LN229 and U251 cell lines were infected with the viral suspension according to the manufacturer's instructions for 48 h before assay.

### Western blot

Total protein from the indicated cell lines was prepared as previously described. Glioma tissues were ground into powder in liquid nitrogen, and total protein was extracted from the tissue powder using RIPA buffer.

Nuclear and cytoplasmic proteins from four GBM cell lines were isolated using the Nuclear and Cytoplasmic Protein Extraction Kit (Beyotime, China) according to the manufacturer's instructions. The protein samples were resolved by SDS-PAGE and transferred onto PVDF membranes (Roche, Basel, Switzerland). The membranes were then incubated with the following antibodies: anti-HOXA9 antibody (Abcam, UK; dilution 1:1, 000), anti-HOXA10 antibody (ThermoFisher, USA; dilution 1:500), anti-HOXA11 antibody (Abcam, UK; dilution 1:1, 000), anti-HOXA13 antibody (Abcam, UK; dilution 1:1, 000), anti-β-Catenin antibody (Invitrogen, USA; 0.5 μg/ml), anti-phospho-β-Catenin antibody(Cell Signaling Technology, USA; at 0.5 μg/ml), anti-SMAD2 antibody (Invitrogen, USA; 1 μg/ml), anti-phosphp-SMAD2 antibody (Invitrogen, USA; at 1 μg/ml), anti-SMAD3 antibody (Invitrogen, USA; 1 μg/ml), or anti-phosphp-SMAD3 antibody (Abcam, UK; dilution 1:1, 000). Antibodies against Histone H3 (Ray Antibody Biotech, China; dilution 1:2000) or GAPDH (ZSGB-BIO, China; dilution 1:2000) were used as normalization and loading controls. The membrane was then incubated with a horseradish peroxidase-conjugated secondary antibody (ZSGB-BIO, China; dilution 1:2000). Immunoreactive proteins were detected using an ECL Western Blotting Substrate (Fisher Scientific).

### Quantitative real-time PCR

Total RNA from the cell lines and frozen and homogenized glioma samples was extracted using TRIzol reagent (Life Technologies). Single-stranded cDNA was synthesized using the GoScriptTM Reverse Transcription System (Promega, USA), starting with 1 μg of RNA, according to the manufacturer's protocol. To detect mRNA expression, quantitative reverse transcription-polymerase chain reaction (qRT-PCR) was performed using a Reverse Transcription System (Promega) according to the manufacturer's instructions using a CFX96 Real-Time PCR Detection System (Bio-Rad Laboratories).

### Immunofluorescence analysis

Cultured U87, U87 EGFRvIII, LN229, and U251 cells were fixed with 4% paraformaldehyde in PBS (Santa Cruz Biotechnology, USA) for 20 min at room temperature. Thereafter, the cells were permeabilized with 0.2% Triton X 100 in PBS for 15 min. Non-specific binding was blocked by incubation with 5% goat serum in PBS for 30 min. The following primary antibodies used for immunofluorescence: anti-HOXA13 antibody (Abcam, UK; dilution 1:100), anti-EGFR antibody (Invitrogen, USA; 2 μg/ml), anti-phosphpo-SMAD2 antibody (Invitrogen, USA; 2 μg/ml), anti-SMAD3 antibody (Invitrogen, USA; at concentration of 2 μg/ml), anti-SMAD2 antibody (Invitrogen, USA; 2 μg/ml), and anti-phosphpo-SMAD3 antibody (Abcam, UK; dilution 1:100). The slides were then incubated in the appropriate antibodies in antibody dilution buffer overnight at 4°C, followed by a further incubation at room temperature for 1 h with Alexa Fluor^®^488 donkey anti-mouse IgG antibody (Invitrogen, USA; 1:500), Anti-rabbit IgG Fab2 Alexa Fluor^®^488 antibody (Cell Signaling Technology, USA; dilution 1:500) or Alexa Fluor^®^594 donkey anti-rabbit IgG antibody (Invitrogen, USA; 1:500). The samples were washed and embedded into mounting medium with 4, 6-diamino-2-phenylindole (DAPI; nuclear DNA was labeled in blue; VECTOR, USA). Microscopy analysis was performed using a confocal laser scanner microscope.

### Cell biological functions assays

#### 3-(4, 5-dimethyl-2-thiazolyl)-2, 5-diphenyl-2-H-tetrazolium bromide (MTT) assay

Cell growth rates were measured using MTT assays. The indicated cells were plated in 96-well plates and transfected with lentivirus. Following treatment, each well was incubated with 20 μL of 5 mg/mL 3-(4, 5-dimethylthiazol-2yl)-2, 5-diphenyl-tetrazolium bromide (MTT) for 4 h in a CO_2_ incubator at 37°C. The medium was aspirated, and 0.2 mL DMSO was added per well. The proliferation rates were measured via colorimetric assays of formazan intensity in a plate reader at 490 nm.

#### Trans well assay

The invasive capacities of human GBM cells were measured using *in vitro* invasion assays (Becton Dickinson Bio-Coat Matrigel Invasion Chamber). The cells were seeded in serum-free DMEM in the upper chambers of each well of a 24-well Matrigel-coated invasion plate. The cells were induced to invade toward a chemo-attractant (5% FBS in DMEM) that was placed into the lower chambers of the wells. After a 24 h incubation, the non-invading cells were removed from the upper surfaces of the invasion membranes, and the cells on the lower surface were stained with crystal violet. The average number of cells per field was determined by counting the cells in 6 random fields per well. Images from each well were captured by microscopic analysis using a camera-equipped Olympus Vanox microscope. All experiments were performed in triplicate.

#### Apoptosis assay

Annexin V/PI staining was performed to quantify apoptosis. Glioma cells in the log growth phase were collected and subjected to Annexin V/PI staining using an Annexin V-FITC Apoptosis Detection Kit (BioVision, Palo Alto, CA) according to the manufacturer's protocol. The resulting fluorescence was measured by flow cytometry using a FACS flow cytometer. The obtained data were analyzed using FlowJo software.

#### Cell cycle distribution

U87, U87-EGFRvIII, LN229, and U251 cells (1 × 10^5^ cells) were plated in 60-mm culture plates, and the cells were treated as previously described. After 2 days, the cells were trypsinized, fixed in 70% ethanol, washed once with PBS, and then labeled with propidium iodide (Sigma-Aldrich) in the presence of RNase A (Sigma-Aldrich) for 30 min in the dark (50 g/mL). Samples were run on a FACScan flow cytometer (Becton-Dickinson, FL, NJ, USA), and the percentage of cells within each phase of the cell cycle was analyzed using FlowJo software.

### Nude mouse glioma intracranial model

BALB/c-A nude mice at 3–4 weeks of age were purchased from the Animal Center at the Cancer Institute at Chinese Academy of Medical Science (Beijing, PR China). A total of 0.5 × 10^5^ U87 EGFRvIII glioblastoma cells transduced with Lenti-NC or Lenti-si HOXA13 virus were implanted stereotactically to establish intracranial gliomas using cranial guide screws. The mice were imaged for Fluc activity using a bioluminescence imaging system (Caliper IVIS Spectrum, USA) on days 7, 14 and 21 after implantation. Bioluminescence imaging was used to detect intracranial tumor growth as previously described. The Living Images software package (Caliper Life Sciences) was used to determine the integrated flux of photons (photons per second) within each interesting region. The data were normalized to the bioluminescence at the initiation of treatment for each animal. The error bars shown in the figures indicate the standard deviation (SD). The mice were sacrificed after observation. Their brains were extracted and fixed in 10% formalin for 24 hours and then embedded in paraffin for H&E and IHC.

### Hematoxylin-eosin (H&E) staining and immunohistochemistry analysis

The paraffin-embedded tissue sections were used for H&E staining and the examination of Ki67 and HOXA13 expression. Sections were dewaxed, treated with 3% H_2_O_2_ for 10 min, incubated with the appropriate primary antibodies (1:100; Santa Cruz Biotechnology) overnight at 4°C, and then treated with a biotinylated secondary antibody (1:100) for 1 h at room temperature. After washing with PBS, the sections were incubated with 3, 3′-diaminobenzidine (DAB) for approximately 1 min, rinsed in PBS, counterstained with hematoxylin, and visualized using a microscope.

### Statistical analysis

SPSS version 18.0 was used for all statistical analyses. The *t*-test was used to determine differences in each 2-group comparison. One-way ANOVA was used to test for differences between at least 3 groups, and a least significant difference post-hoc test was used to obtain individual *P*-values followed by ANOVA. Differences in survival were assessed using the Kaplan-Meier method and analyzed using the log-rank test in univariate analysis. To assess the relative risk for each factor, univariate and multivariate Cox regression analysis were performed. The median value was used as a cut-off score to discriminate between high and low HOXA13 expression. KEGG pathway and GO analysis was performed using DAVID (http://david.abcc.ncifcrf.gov/). Heat maps were constructed using Gene Cluster 3.0 and Gene Tree View software. A two-sided *p*-value of < 0.05 was regarded as significant. Gene Set Enrichment Analysis (GSEA) was used to determine whether there was a statistically significant difference in the genes between the high HOXA13 and low HOXA13 conditions. To determine the differential genes associated with HOXA13, Matlab software was used to perform Pearson Correlation. All data are presented as the mean ± standard error. All tests were two-sided, and *p*-values < 0.05 were considered to be statistically significant.

## References

[1] Qian YQ, Billeter M, Otting G, Muller M, Gehring WJ, Wuthrich K (1989). The structure of the Antennapedia homeodomain determined by NMR spectroscopy in solution: comparison with prokaryotic repressors. Cell.

[2] Lewis EB (1978). A gene complex controlling segmentation in Drosophila. Nature.

[3] Akam M (1998). Hox genes: from master genes to micromanagers. Curr Biol.

[4] Noordermeer D, Leleu M, Splinter E, Rougemont J, De Laat W, Duboule D (2011). The dynamic architecture of Hox gene clusters. Science.

[5] Klein D, Benchellal M, Kleff V, Jakob HG, Ergün S (2013). Hox genes are involved in vascular wall-resident multipotent stem cell differentiation into smooth muscle cells. Scientific Reports.

[6] Gehring WJ, Hiromi Y (1986). Homeotic genes and the homeobox. Annu Rev Genet.

[7] Kappen C, Schughart K, Ruddle FH (1989). Organization and expression of homeobox genes in mouse and man. Ann N Y Acad Sci.

[8] Quagliata L, Matter MS, Piscuoglio S, Arabi L, Ruiz C, Procino A, Kovac M, Moretti F, Makowska Z, Boldanova T, Andersen JB, Hämmerle M, Tornillo L, Heim MH, Diederichs S, Cillo C (2014). Long noncoding RNA HOTTIP/HOXA13 expression is associated with disease progression and predicts outcome in hepatocellular carcinoma patients. Hepatology.

[9] Bhatlekar S, Fields JZ, Boman BM (2014). HOX genes and their role in the development of human cancers. Journal of Molecular Medicine.

[10] Richards EJ, Zhang G, Li ZP, Permuth-Wey J, Challa S, Li Y, Kong W, Dan S, Bui M, Coppola D, Mao WM, Sellers TA, Cheng JQ (2015). Long non-coding RNAs regulated by TGF-β: lncRNA-HIT mediated TGFbeta-induced epithelial to mesenchymal transition in mammary epithelia. J Biol Chem.

[11] Costa BM, Smith JS, Chen Y, Chen J, Phillips HS, Aldape KD, Zardo G, Nigro J, James CD, Fridlyand J, Reis RM, Costello JF (2010). Reversing HOXA9 oncogene activation by PI3K inhibition: epigenetic mechanism and prognostic significance in human glioblastoma. Cancer Res.

[12] Murat A, Migliavacca E, Gorlia T, Lambiv WL, Shay T, Hamou MF, de Tribolet N, Regli L, Wick W, Kouwenhoven MC, Hainfellner JA, Heppner FL, Dietrich PY, Zimmer Y, Cairncross JG, Janzer RC (2008). Stem cell-related “self-renewal” signature and high epidermal growth factor receptor expression associated with resistance to concomitant chemoradiotherapy in glioblastoma. J Clin Oncol.

[13] Gaspar N, Marshall L, Perryman L, Bax DA, Little SE, Viana-Pereira M, Sharp SY, Vassal G, Pearson AD, Reis RM, Hargrave D, Workman P, Jones C (2010). MGMT-independent temozolomide resistance in pediatric glioblastoma cells associated with a PI3-kinase-mediated HOX/stem cell gene signature. Cancer Res.

[14] Zhang JX, Han L, Bao ZS, Wang YY, Chen LY, Yan W, Yu SZ, Pu PY, Liu N, You YP, Jiang T, Kang CS (2013). HOTAIR, a cell cycle-associated long noncoding RNA and a strong predictor of survival, is preferentially expressed in classical and mesenchymal glioma. Neuro-Oncology.

[15] Zhang K, Sun X, Zhou X, Han L, Chen L, Shi Z, Zhang A, Ye M, Wang Q, Liu C, Wei J, Ren Y, Yang J (2015). Long non-coding RNA HOTAIR promotes glioblastoma cell cycle progression in an EZH2 dependent manner. Oncotarget.

[16] van den Bent M, Chinot OL, Cairncross JG (2003). Recent developments in the molecular characterization and treatment of oligodendroglial tumors. Neuro-Oncology.

[17] Kalpathy-Cramer J, Gerstner ER, Emblem KE, Andronesi OC, Rosen B (2014). Advanced Magnetic Resonance Imaging of the Physical Processes in Human Glioblastoma. Cancer Res.

[18] Pan T, Jia W, Yao Q, Sun Q, Ren W, Huang M, Ma J, Li J, Ma J, Yu J, Ge Y, Liu W, Zhang C, Xu G (2014). Overexpression of HOXA13 as a Potential Marker for Diagnosis and Poor Prognosis of Hepatocellular Carcinoma. The Tohoku Journal of Experimental Medicine.

[19] Han Y, Tu W, Wen Y, Li D, Qiu G, Tang H, Peng Z, Zhou C (2013). Identification and validation that up-expression of HOXA13 is a novel independent prognostic marker of a worse outcome in gastric cancer based on immunohistochemistry. Medical Oncology.

[20] Zhao J, Zhang L, Guo X, Wang J, Zhou W, Liu M, Li X, Tang H (2015). miR-212/132 downregulates SMAD2 expression to suppress the G1/S phase transition of the cell cycle and the epithelial to mesenchymal transition in cervical cancer cells. IUBMB Life.

[21] He Z, Jiang J, Kokkinaki M, Dym M (2009). Nodal Signaling via an Autocrine Pathway Promotes Proliferation of Mouse Spermatogonial Stem/Progenitor Cells Through SMAD2/3 andOct-4 Activation. Stem Cells.

[22] Sengupta S, Jana S, Bhattacharyya A (2014). TGF-β-SMAD2 dependent activation of CDC 25A plays an important role in cell proliferation through NFAT activation in metastatic breast cancer cells. Cellular Signalling.

[23] Li Z, Zhao X, Zhou Y, Liu Y, Zhou Q, Ye H, Wang Y, Zeng J, Song Y, Gao W, Zheng S, Zhuang B, Chen H, Li W, Li H, Li H (2015). The long non-coding RNA HOTTIP promotes progression and gemcitabine resistance by regulating HOXA13 in pancreatic cancer. Journal of Translational Medicine.

[24] Thiery JP, Acloque H, Huang RYJ, Nieto MA (2009). Epithelial-Mesenchymal Transitions in Development and Disease. Cell.

[25] Song J (2007). EMT or apoptosis: a decision for TGF-beta. Cell Res.

[26] Holtzhausen A, Golzio C, How T, Lee YH, Schiemann WP, Katsanis N, Blobe GC (2014). Novel bone morphogenetic protein signaling through SMAD2 and SMAD3 to regulate cancer progression and development. The FASEB Journal.

[27] Dai F, Duan X, Liang YY, Lin X, Feng XH (2010). Coupling of dephosphorylation and nuclear export of SMADs in TGF-beta signaling. Methods Mol Biol.

[28] Massague J (2005). SMAD transcription factors. Genes & Development.

[29] Liu S, Jin K, Hui Y, Fu J, Jie C, Feng S, Reisman D, Wang Q, Fan D, Sukumar S, Chen H (2015). HOXB7 promotes malignant progression by activating the TGFbeta signaling pathway. Cancer Res.

[30] Williams TM (2005). Group 13 HOX proteins interact with the MH2 domain of R-SMADs and modulate SMAD transcriptional activation functions independent of HOX DNA-binding capability. Nucleic Acids Research.

[31] Shi Y, Massague J (2003). Mechanisms of TGF-beta signaling from cell membrane to the nucleus. Cell.

[32] Clevers H, Nusse R (2012). Wnt/β-Catenin Signaling and Disease. Cell.

[33] Shi Z, Qian X, Zhang J, Han L, Zhang K, Chen L, Zhou X, Zhang J, Kang C (2014). BASI, A Potent Small Molecular Inhibitor, Inhibits Glioblastoma Progression by Targeting microRNA-mediated. CNS Neuroscience & Therapeutics.

[34] Shi Z, Qian X, Liu C, Han L, Zhang K, Chen L, Zhang J, Pu P, Yuan X, Kang C (2013). Aspirin-/TMZ-coloaded Microspheres Exert Synergistic Antiglioma Efficacy via Inhibition of β-catenin Transactivation. CNS Neuroscience & Therapeutics.

[35] Zhang K, Zhang J, Han L, Pu P, Kang C (2012). Wnt/beta-Catenin Signaling in Glioma. Journal of Neuroimmune Pharmacology.

